# Metagenomic sequencing reveals the relationship between microbiota composition and quality of Chinese Rice Wine

**DOI:** 10.1038/srep26621

**Published:** 2016-05-31

**Authors:** Xutao Hong, Jing Chen, Lin Liu, Huan Wu, Haiqin Tan, Guangfa Xie, Qian Xu, Huijun Zou, Wenjing Yu, Lan Wang, Nan Qin

**Affiliations:** 1 Zhejiang-California International Nanosystem Institute, Zhejiang University, 866 Yuhangtang Road, Hangzhou 310058, China; 2 College of Life Sciences, Zhejiang University, Hangzhou 310058, People’s Republic of China; 3 Realbio Genomics Institute, Shanghai, 200050, China; 4 State Key Laboratory for Diagnosis and Treatment of Infectious Disease, The First Affiliated Hospital, College of Medicine, Zhejiang University, Hangzhou, 310003, China; 5 Collaborative Innovation Center for Diagnosis and Treatment of Infectious Diseases, Zhejiang University, Hangzhou, 310003, China; 6 National Engineering Research Center for Chinese Rice Wine, Zhejiang Guyuelongshan Shaoxing Rice Wine Co.Ltd., Shaoxing, Zhejiang 312000, China

## Abstract

Chinese Rice Wine (CRW) is a common alcoholic beverage in China. To investigate the influence of microbial composition on the quality of CRW, high throughput sequencing was performed for 110 wine samples on bacterial 16S rRNA gene and fungal Internal Transcribed Spacer II (ITS2). Bioinformatic analyses demonstrated that the quality of yeast starter and final wine correlated with microbial taxonomic composition, which was exemplified by our finding that wine spoilage resulted from a high proportion of genus *Lactobacillus*. Subsequently, based on *Lactobacillus* abundance of an early stage, a model was constructed to predict final wine quality. In addition, three batches of 20 representative wine samples selected from a pool of 110 samples were further analyzed in metagenomics. The results revealed that wine spoilage was due to rapid growth of *Lactobacillus brevis* at the early stage of fermentation. Gene functional analysis indicated the importance of some pathways such as synthesis of biotin, malolactic fermentation and production of short-chain fatty acid. These results led to a conclusion that metabolisms of microbes influence the wine quality. Thus, nurturing of beneficial microbes and inhibition of undesired ones are both important for the mechanized brewery.

Chinese Rice Wine (CRW), including some acclaimed local varieties produced in Shaoxing, Zhejiang Province, is an alcoholic beverage fermented using rice and wheat. As a traditional alcoholic drink, CRW has been popular in China for centuries and honored as a national banquet wine^1^. The traditional CRW is brewed from glutinous rice and wheat, fermented by wheat Qu and yeast starter^2^. Wheat Qu is made from wheat and harbors a variety of microorganisms, such as *Aspergillus*, *Rhizopus*, *Mucor*, *Absidia*, *Lactobacillus*, *Bacillus*, and *Acetobacteria*^3^,^4^, which can produce a plethora of enzymes contributing to starch breakdown and subsequent alcohol generation. Yeast starter mainly contains *Saccharomyces cerevisiae* and a few bacterial species, of which *S. cerevisiae* is the main source of alcohol production. When yeast starter is mixed with wheat Qu under appropriate temperature and humidity conditions, fermentation is initiated that ultimately culminates in producing of specially flavored beverage. As such, many microorganisms play indispensable roles in CRW production and studies on the relevant microbial composition may help to produce high-quality wines.

Most studies on the influence of microbial composition on CRW quality depended on plate cultivation methods^5^,^6^, which may be limited by the cultivability of some species. Currently, only a few microbial species were identified, such as strains of *Saccharomyces*, *Aspergillus*, *Bacillus*, and *Lactobacillus*. Another option to evaluate microbial composition is denaturing gradient gel electrophoresis (PCR-DGGE), which however is expensive and time-consuming and has limited ability to detect rare or non-cultivable microbes^7^,^8^.

Since its emergence, the technology of next generation sequencing (NGS) has been used to elucidate the structure of the microbial community of many kinds of biotopes, such as soil, seawater and food^9^,^10^,^11^. In the present study, we performed high-throughput metagenomic sequencing to examine 110 CRW samples in the V3–V4 regions of the bacterial 16S rRNA gene as well as fungal ITS2 region. The test samples contained rice wine samples of different fermentative phases and several yeast starters. Subsequently, we performed a complete metagenomic study for three representative batches of 20 CRW samples. The analyses enabled us to uncover the composition of the bacterial and fungal communities of CRW on species level. Moreover, gene function analysis was also performed, which revealed some critical genetic factors for CRW making. Together, this study might provide valuable microbiota knowledge for quality control during CRW production.

## Results

### 16S/ITS amplicon sequencing of yeast starter and CRW

The Illumina Miseq platform was employed to sequence the V3–V4 regions of 16S rRNA gene of 109 samples (14 samples from yeast starter and 95 from 14 batches of wines in fermentation), thereby obtaining 8.04 million clean tags (73,749 ± 20,507 on average) after concatenation and quality control. Of all test samples, 108 ones produced 3.89 million clean ITS2 tags (36,039 ± 25,204 per sample). One sample failed in sequencing library construction in 16S, as did two in ITS2. Sample statistic information was listed in the Supplementary Table 1.

#### Association between microbial composition of 16S/ITS amplicon and quality of yeast starter and CRW

**Figure 1:**
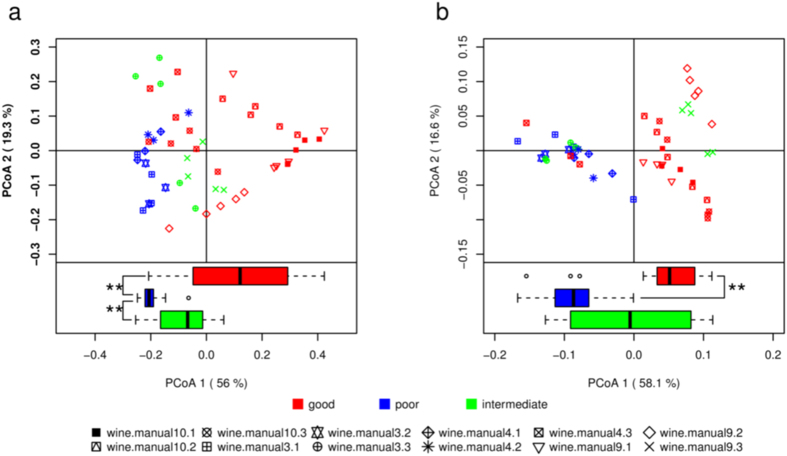
Bacterial and fungal composition on genus level differs between wine samples of good and poor qualities. Only samples fermented for at least 7 days were examined. (**a**) PCoA of 16S genus. The shorter the fermentation time is, the more the PCoA1 is possible to be larger within the samples of the same batch. (**b**) PCoA of ITS genus. The differences between boxes were tested by Wilcoxon test (**≤0.01). Monte Carlo permutation test also shows a significant separation between groups of wines with p value ≤ 0.001 for 999 repeats. Points of the same shape belong to the same batch of samples in fermentation.

To elucidate whether there was a connection between microbial composition and CRW quality, principal coordinate analysis (PCoA) was performed for 12 batches of traditional manual wine samples labeled with quality information (Supplementary Table 2), based on the abundance profiling of bacteria and fungi on the genus level. This allowed us to examine whether taxonomic composition displayed a significant correlation with wine quality. It was observed that the differences between wines of good and poor qualities was not clear in the first several days but became increasingly pronounced as the fermentation proceeded. Hence, wine samples with a fermentation period less than 7 days were not included in the PCoA analyses. As shown in both panels of PCoA in Fig. 1, the first dimension of PCoA1 generally separated good wines from poor ones well. However, the coordinates of the good quality batch “wine.manual4.3” appeared to be in the vicinity of poor quality samples. One explanation was that all the “wine.manual4.3” samples were extracted in the first 30 days of fermentation, but it normally required 70 days for all the process of traditional manual fermentation. The results also imply that some fermenting processes might be relatively slow. In the case of yeast starter, samples were also well separated by qualities (Supplementary Fig. 1). The data collectively indicated that microbial composition is a key factor for final wine quality.

#### Relative abundance of *Lactobacillus* on the 7^th^ day of fermentation presaged final wine quality

**Figure 2:**
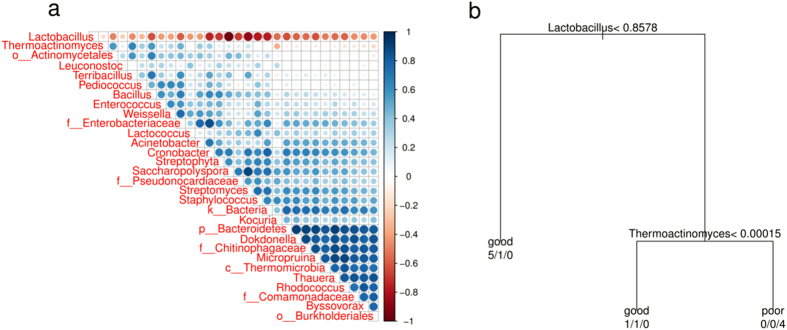
*Lactobacillus* in the samples of 7^th^ day presaged final quality of wines. (**a**) Correlation plot shows beneficial and antagonistic relationships between genera of 16S. The prefixes “k__”, “p__”, “c__”, “o__”, “f__” indicate OTUs only annotated to the level of kingdom, phylum, class, order or family. Correlation was expressed by Spearman correlation coefficient. Only the first 30 abundant genera were analyzed. (**b**) Decision tree based on samples of 7^th^ day of fermentation. The abundances of “*Lactobacillus*” and “*Thermoactinomyces*” were different enough to distinguish successful fermentation from failed one.

As the PCoA analyses revealed differences of taxonomic composition between wines of good and poor qualities (Fig. 1), we were interested in whether the wine quality was predictable by taxonomic composition of the relevant preceding samples. Wine spoilage often results from furious growth of one undesired strain at the beginning of fermentation^12^. Once this strain becomes dominant, it inhibits other microbes including many beneficial ones, leading to fermentation failure. Thus, we attempted to find beneficial or antagonist relationships between different microbial groups on the genus level. As shown in Fig. 2a, one apparent pattern was that *Lactobacillus* appeared antagonist to all of the rest genera. Indeed, *Lactobacillus* was almost always the most abundant genus over the course of fermentation (Supplementary Fig. 2) and its abundance varied relatively little between samples in the same batch. It has been reported that some wine spoilages were associated with *Lactobacillus*^13^. Several species of *Lactobacillus* are lactic acid bacteria (LAB), which convert carbohydrates to lactic acid in homofermentation or to lactic acid and acetic acid/ethanol in heterofermentation. Production of the two acids acidifies the environment and inhibits the growth of other microbes. Moreover, acetic acid interferes with and slows the fermentation of *Saccharomyces*^14^. Thus, it seems reasonable to predict final wine quality using *Lactobacillus* percentage over the course of fermentation.

As illustrated in Fig. 2b, a decision tree was established mainly based on *Lactobacillus* abundance along with *Thermoactinomyces* abundance which also revealed some weak antagonism with some microbes. The deviation of the abundance of these two genera occurred as early as on the 7^th^ day of the fermentation, which therefore was the earliest time-point to predict the final wine quality. On this day, a low ratio (≤0.8578) of *Lactobacillus* was always associated with good fermentation, whereas relatively high ratios of *Lactobacillus* and *Thermoactinomyces* inevitably resulted in failed fermentation.

### Taxonomic analysis in metagenomics

To identify the genetic elements and species affecting wine quality, 20 wine samples, which were in different fermentation stages and belonged to three batches of wines, were interrogated by analyses of metagenomics. DNA of eukaryotic and prokaryotic origins was extracted, fragmented and sequenced by Illumina Hiseq 2500, which generated a total of 136 Gbp of raw reads of PE125 (6.78 ± 2.51 Gbp on average, Supplementary Table 3). However, there were averagely only 3.87 ± 1.73 Gbp left after quality control, because about 42% of reads originated from cereals used in fermentation. Experimental extraction of DNA could not separate fungal DNA from cereal DNA, leading to a high proportion of contaminating reads. The clean reads were then aligned to fungal and bacterial genomes downloaded from NCBI and HMP. Approximately 18.9% of the clean reads could map to these genomes, much less than previously reported 31% in human gut metagenome^15^. This can be explained by a possibility that there are still many genomically unstudied microbes in the environment, whereas gut microbes are relatively better studied and sequenced.

#### Genome-dependent taxonomic composition in metagenomics

Metagenomics-based taxonomic composition was then compared with that calculated from the16S and ITS2 sequences on genus level (Supplementary Fig. 3). Interestingly, although the two types of fungal compositions (i.e., metagenomics-based and ITS2-based) were extremely similar to each other (Supplementary Fig. 3c,d), the 16S-based bacterial compositions showed some differences from the metagenomics-based ones (Supplementary Fig. 3a,b). These differences in bacterial taxonomic compositions was mainly caused by the inherent precision or bias of the two sequencing approaches^16^,^17^,^18^; the discrepancy between metagenomics-based and 16S-based approaches have been reported previously^19^,^20^,^21^. Nevertheless, these results collectively revealed the great microbial diversity of the fermentation samples.

#### Lactobacillus brevis caused spoilages

Metagenomic taxonomic analyses were performed to identify the very species responsible for spoilage and to reveal the metabolic differences between good and poor fermentations. The zymotic fluid of wine can be viewed as a microbial eco-system in which each species metabolized different substrates and produced different compounds, which makes the fermentation process complex and the resulting flavors distinct.

**Figure 3:**
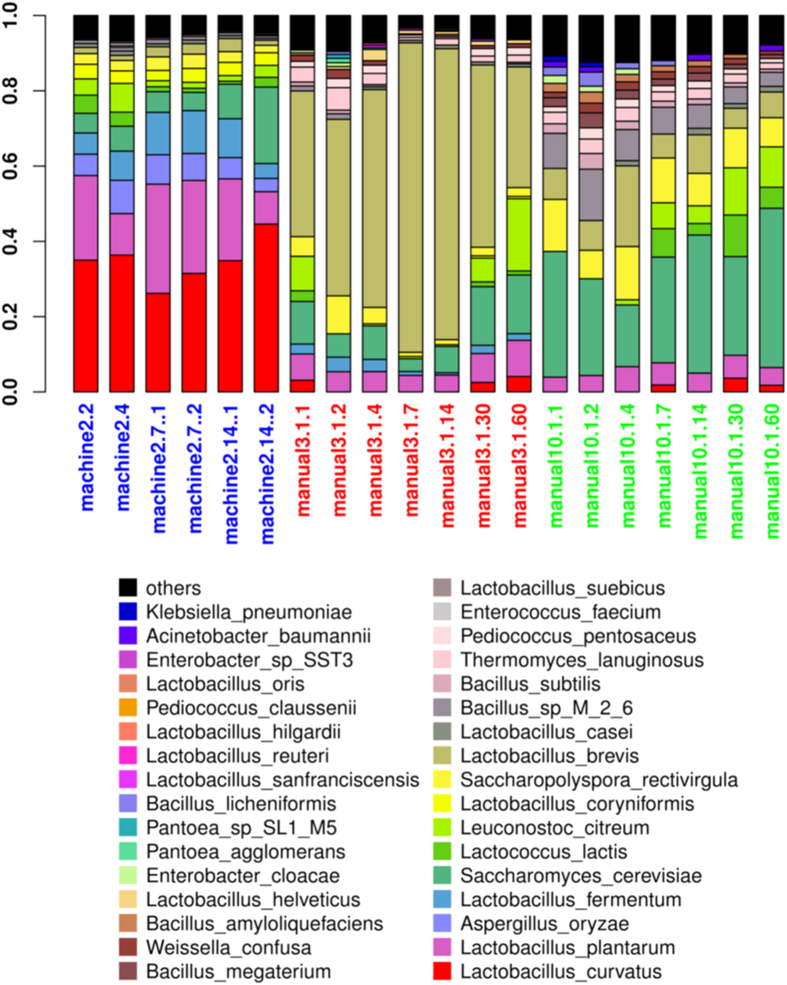
Bacterial and fungal composition of metagenomic samples on species level. Samples are sorted based on the batches and time of fermentation. Each bar shows top15 microbes. The samples prefixed by “machine2” come from mechanized fermentation, while the “manual” ones from traditional manual fermentation. The wines, prefixed by “manual3.1” belonged to a failed fermentation and the ones with prefix of “manual10.1” were from a successful fermentation. (Detailed methods of naming samples are included in Supplementary Table 1).

The results of metagenomic taxonomic composition on species level were shown in Fig. 3. Globally, microbial composition was more stable in wines of mechanized production than in traditional manual wines, because some environmental parameters, such as temperature and humidity, were more standardized and controlled in mechanized fermentation than in traditional manual brewery. Samples with the suffixed “..1” were extracted from supernatant and were close to samples of the same time point from turbid liquid, which were suffixed with “..2”, on the 7^th^ day of mechanized fermentation. However, differences appeared on the 14 days between supernatant and turbid liquid. Globally, species compositions were very different between the three batches. *Lactobacillus* had the highest number of species and was also very abundant. *L. plantarum* and *L. curvatus* accounted for high percentages in the mechanized samples prefixed by “machine2”, while in the failed manual samples prefixed by “manual3.1”, *L. brevis* alone could account for as much as 80% of the microbial abundance. These three heterofermentative *Lactobacillus* species accounted for a high portion in both batches of “machine2” and “manual3.1”, but the qualities of the two batches were apparently different. This may be correlated to the fact that whereas both *L. plantarum* and *L. curvatus* are facultatively heterofermentative, *L. brevis* is obligately heterofermentative. It was reported that *L. brevis* caused failed fermentation in beer^13^ and red wine and that the microbe synthesized compounds leading to offensive odors and unpleasant taste^22^. On the other hand, *L. plantarum* seemed to be one factor for the good quality of the mechanized wines, because this bacterium can perform malolactic fermentation (MLF)^23^, in which malic acid is metabolized to lactic acid to decrease wine acidity^24^. Moreover, it also secrets bacteriocins which have antibacterial activity^25^ that may help to establish its dominance to favor fermentation. The “manual10.1” samples were enriched in fungi including *S. cerevisiae* and *Saccharopolyspora rectivirgula*, consistent with a previous finding that red wine fermentation requires a dominance of *S. cerevisiae*^26^. Moreover, mechanized fermentation proceeded much faster than traditional manual fermentation, which might be due to *Aspergillus Oryzae*. *A. Oryzae* has strong secretion of amylases including alpha-amylase, which will certainly accelerate the degradation of grains and provide more nutrients for microbes in fermentation. Li, H. *et al.*^27^ reported an improvement of rice wine fermentation using grinded grains, suggesting the importance of quick grain degradation in the course of rice wine fermentation. The results here revealed that different microbial makeup greatly influenced the fermentation process.

#### *L. brevis* caused spoilage or made fermentation sluggish

**Figure 4:**
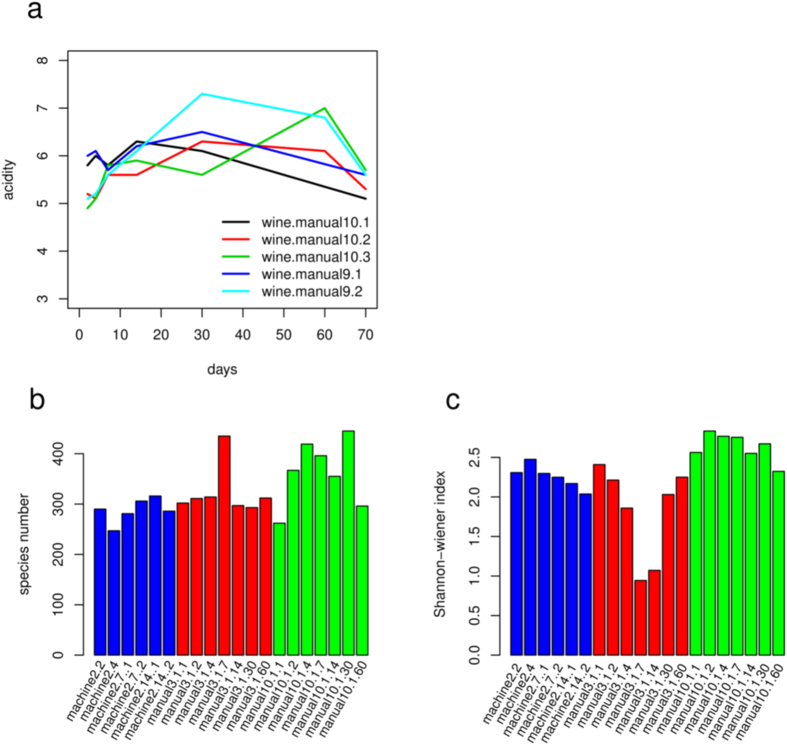
Variation of acidity, species number and microbial evenness over times. (**a**) Acidity changed over the course of fermentation. The acidity increased at the beginning and decreased later. (**b**) Species number counting. (**c**) Shannon-wiener index shows the evenness of species.

Many bacteria were reported to slow fermentation in wine industry^28^,^29^. Several phenomena demonstrate the possibility that high ratio of *L. brevis* might cause such a problem. The wine quality is mainly evaluated based on taste, which is influenced by acids as they turn the wine sour. Over the course of a good fermentation, the acid level was the highest in the middle phase of fermentation (Fig. 4a), suggesting that wines with high acidity might not have been incubated for enough time. Figure 3 showed that from 14 to 60 days post inoculation, the Shannon-wiener index, reflecting the evenness of species composition increased (Fig. 4c) and the microbial composition of “manual3.1” had a tendency to match that of “manual10.1”. In addition, *L. brevis* also accounted for a sizable proportion in “manual10.1” of good quality, suggesting that *L. brevis* possibly was not harmful to other microbes important for fermentation if not at a dominant level. Thus, inhibition of other microbes by *L. brevis* seemed to be manageable.

### Gene function analysis in metagenomics

With such taxonomic differences observed, we were interested in which metabolic pathways could influence the wine quality. Therefore, metagenomic protein sequences were aligned to different databases in which the function of each protein sequences had been classified, which helped us to study metabolic differences in each sample. The different aspects between good and poor fermentation were possibly the signs to which we should pay attention.

#### QS (Quorum sensing) analysis

**Figure 5:**
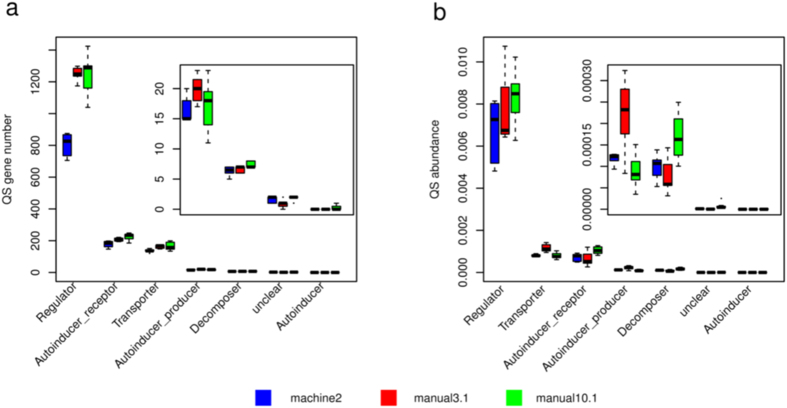
QS-related gene number and abundance differ between batches.

The zymotic fluid contained high abundance of microbes, which can adjust their metabolism to adapte to the adverse environment (e.g. low pH, high alcohol concentration) as well as inter-bacterial competition. For example, bacteria excrete bacteriocins to inhibit other microbes or detect virulence and respond accordingly to survive. Many of these activities are under the control of quorum sensing (QS) systems. Metagenomic proteins were annotated to QS-related proteins. The statistic results of gene number and gene abundance were illustrated in Fig. 5. Tranditional manual fermentation had more QS genes of “Regulator”. This might be attributable to a possibility that there were more species in traditional manual wines samples than in mechanized samples (Fig. 4b). For the batch of poor quality “manual3.1”, there were more genes for autoinducer transport and autoinducer sythesis. Considering that *L. brevis* was the most abundant species in the failed fermentation, this might simply reflect the high abundance of *L*. *brevis* that harbors QS genes. In contrast, the good batch “manual10.1” had more genes for autoinducer regulation and quorum inhibition such as quorum-quenching enzymes, which suggested that the species in good fermentation might be more independent. Together, QS-based communication bewteen microbes in zymotic fluid influenced wine fermentation.

#### AR (Antibiotic Resistance) gene abundances were significantly higher in traditional manual wines

As mentioned above, many microbes coexisted in zymotic fluid, which aggravated the competition for nutrients. In order to compete, some species might produce a variety of antibiotics or bacteriocins to inhibit the growth of other microbes. Analyzing AR genes could indicate the expression level and the strength of antibiotics.

Supplementary Fig. 4a illustrated that AR genes were apparently more abundant in good quality wines. The tendencies of total AR gene abundances were clearly different between wines of good and poor qualities during the first several days. Supplementary Fig. 4b shows that the most abundant AR type was “baca”, but “baca” and “nora” were more predominant in mechanized samples. In the “manual3.1” samples of poor quality, “acrb” was also concentrated in addition to “baca”. However, in the “manual10.1” samples of good qualify, several AR types were enriched. Day 7 of fermentation appeared to be a time point for transitioning of fermentation stage, because the abundance of AR types (lmrp, emea, tets) increased and the types (nora, acra, lnua ksga, mdfa, bl2a_iii2, *etc.*) decreased on this day. However, the AR gene abundances were relatively stable over the course of fermentation in the batch “manual3.1”. This supported our previous model using the 7^th^ day for the earliest quality prediction and also agreed with our hypothesis that *L. brevis* caused a sluggish fermentation. AR gene analysis therefore suggested that regulating the microbial composition might suppress the growth of undesirable microbes during fermentation.

#### KEGG annotation exposes metabolic differences between good and poor fermentation

**Figure 6:**
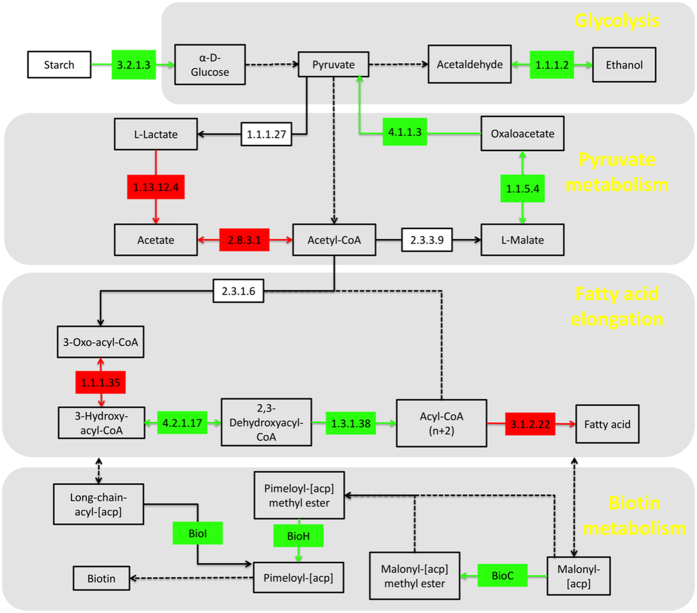
Summarized KEGG pathways. Genes, significantly abundant in “manual10.1.7”, were annotated in green, while these, abundant in “manual3.1.7”, were annotated in red. The solid lines correspond to only one reaction on KEGG pathway, while the dotted lines mean more than one reaction.

Markers genes that were significantly enriched in the samples of “manual10.1.7” or “manual3.1.7” were annotated on KEGG pathway (Fig. 6). Starch, which was the main component in raw material of wine fermentation, might be easier to be hydrolyzed to monosaccharides in “manual10.1.7”. Ethanol-producing genes were also abundant in “manual10.1.7”. In the “manual3.1.7”, pyruvate was more likely to be metabolized to acetate which acidified the zymotic fluid. By contrast, in “manual10.1.7”, malic acid (malate), more acidic than acetic acid, was likely catalyzed to become lactate by MLF. MLF not only converts malic acid into lactic acid, but also produces some substances enhancing wine flavors^30^. The metabolism of acids would directly influence the taste of wine. Two ECs “4.2.1.17” and “1.3.1.38”, enriched in “manual10.1.7”, were responsible for fatty acid elongation and potentially produce medium chain fatty acid which helps MLF in low level and inhibition of bacteria^31^. Three enzymes (“bioC”, “bioH” and “bioI”) were enriched in “manual10.1.7”. These three enzymes are in the pathway of biotin synthesis and biotin has been reported to enhance alcohol fermentation of *S. cerevisiae*^32^.

## Discussion

This study revealed that microbial taxonomic composition apparently differed between high and low quality wines. The spoilage of CRW arose from dominant growth of some species of *Lactobacillus* (e.g., *L. brevis*) in the early stage of fermentation. As such, final wine quality is predictable by samples as early as on the 7^th^ day of fermentation using the relative abundance of *Lactobacillus*. An early prediction of wine quality will help the wine factory to save time and reduce the loss. Identification of undesired species can remind the factory to take measures to augment the growth of *S. cerevisiae*. For example, chemical antimicrobial agents, such as SO_2_, could be employed to inhibit undesired microbes.

Even though the taxonomic compositions were different between samples of “wine.machine2” and “wine.manual10.1”, they were both good quality wines. This indicated that only a few species, such as *S. cerevisiae*, can promote desirable fermentation. The fast rate in mechanized fermentation could suggest that under such environment, growth of the brewery-beneficial species was encouraged. Improving the fermentation condition to expedite the process is always an important task.

Abundances of QS and AR genes were much different between good and poor quality wines. QS analysis revealed that microbes in poor quality wines might have greater level of communications than these in good wines. The result of AR genes displayed a transition phase at around 7^th^ day in good fermentation, while AR genes in poor fermentation relatively remained stable. Whether the stability resulted from a good QS-related communication and therefore strong adaptation needs to be studied. Inoculation of some species on 7^th^ day may help the fermentation to enter next stage.

KEGG results were consistent with previous reports of other types of wines. “manual10.1” samples harbored relatively high abundance of genes promoting wine fermentation and flavors; on the contrary, “manual3.1” had more genes associated with acetate production that suppresses fermentation. In the zymotic fluid of “manual10.1”, there were more vitamins, such as biotin. These metabolism differences and especially consequent compounds possibly influence fermentation and thus help us to upgrade the fermentation process.

In summary, we confirmed the dependence of CRW quality on microbial composition over the course of fermentation after taxonomic analysis. Exceptionally high ratio of *L. brevis* caused wine spoilage that was possible to be a factor for fermentation delay. Subsequently, we succeeded in establishing a model to predict final wine quality using the bacterial composition on 7^th^ day. The functional analyses indicated some potentially important aspects for CRW fermentation which helps brewery to improve the mechanized processes.

## Methods

### Sample collection and storage

At the beginning of the fermentation, a same amount of yeast starters or wheat Qu was added into each fermentation sample. The compositions of the yeast starters and wheat Qu were different for the wine manual and the wine machine. For the manual wine, a naturally cultured yeast starter and wheat Qu were used as the starter inoculum and the saccharification agent respectively. For the mechanized wine, the purely cultured yeast, same as the above, was used as the starter inoculum, while a mixture of the naturally cultured wheat Qu and the wheat Qu inoculated with *Aspergillus oryzae* were used as the saccharification agent.

Samples were collected between February and May, 2011, during the process of manufacturing in a wine-making factory of Shaoxing, Zhejiang Province, China. For the mechanized wine, the supernatant samples and the turbid liquid samples were collected as the follows. At the end of the fermentation, the distillers’ grains were precipitated, and the resulting solution was used as the supernatant. In addition, the distillers’ grains were fully mixed with the supernatant and the mixture used was as the turbid liquid.

After the samples were collected, they were transferred to sterile bottles, sealed, and stored at −80 °C. Samples for bacterial and fungal composition analysis were selected after 8-month storage. The alcohol degree and the acidity assay, measured according to the standard of GB/T13662-2008, were employed to evaluate wine quality. The physiochemical parameters of each CRW sample were showed in the Supplementary Table1.

### DNA extraction

Following the manufacturer’s instructions, genomic DNA was extracted using a commercial Fast DNA SPIN Kit for Soil (MP Biomedicals, USA), before the DNA was used to construct a library.

### 16S/ITS Amplicon analysis

#### Amplification of 16S/ITS tags and library construction

Variable regions V3–V4 on 16S rRNA gene of bacteria and the ITS2 region of fungi were amplified using PCR. Tags of ~420 bp of V3–V4 on 16S rRNA gene were amplified with forward primer 5′-ACTCCTACGGGAGGCAGCAG-3′ and reverse primer 5′-GGACTACHVGGGTWTCTAAT-3′^33^. ITS2 tags of ~350 bp were amplified by forward primer 5′-GCATCGATGAAGAACGCAGC-3′ and 5′-TCCTCCGCTTATTGATATGC-3′^34^. All the tags were barcoded at both ends to distinguish samples and 1~3 nucleotides were inserted between the barcode and the primer to increase nucleotide diversity when performing sequencing reaction^35^. Adaptors were added to complete the construction of libraries. The PCR products were purified with the QIA quick PCR Purification Kit and visualized on the 1% agarose gel and then adjusted to equal concentrations.

#### Illumina Miseq sequencing and reads pre-processing

After preparation of library, these tags were sequenced on Miseq platform for paired end reads of 300 bp, which were overlapped on their 3′ ends for concatenation into original longer tags. Tags, trimmed of barcodes and primers, were further checked on their rest lengths and average base quality. 16S tag was restricted between 350 bp and 500 bp such that the average Phred score of bases was no worse than 20 (Q20) and no more than 3 ambiguous N. The lengths of ITS tags were between 300 and 420 bp.

#### OTU (Operational Taxonomic Unit) picking strategies

We enumerated the copy number of tags and removed redundancy of repeated tags^36^. Only the tags with frequency more than 1, which tend to be more reliable, were clustered into OTUs, each of which had a representative tag. *De novo* chimeric sequences were checked and the entire OTU was abandoned if the representative tag was chimeric. All the initial tags of each sample were mapped to non-chimeric representative tag of OTUs with 0.97 identities, augmenting the number of tags in each OTU. The mapped tags of each sample were randomly downsized to the same number to reduce the difference of depth of sequencing. New representative tags with the most abundance were picked. All the above-mentioned steps were mainly performed by usearch (v7.0.1090_i86linux32) and combined with python script of Qiime and perl scripts.

#### OTU assignment and diversity analysis

Each representative tags was assigned to a taxa by RDP classifier^37^ with assignment confidence cutoff 0.8. Phylogenetic tree was obtained on Qiime pipeline with the method pynast for 16S and muscle for ITS2, respectively. OTU profiling table and alpha/beta diversity analyses were also achieved by python scripts of Qiime.

### Metagenomic sequencing

#### DNA library construction and sequencing

The metagenomic DNA libraries were constructed with 2 μg genome DNA according to the Illumina TruSeq DNA Sample Prep v2 Guide, with an average of 350 bp insert size. The quality of all libraries was evaluated using an Agilent bioanalyser with a DNA LabChip 1000 kit. Sequencing was performed by Illumina Hiseq2500 at WuXi AppTec of China.

#### Quality control of Illumina Hiseq2500 reads

Illumina raw reads were subject to the following treatments: (1) reads with more than 3 ambiguous N bases were removed; (2) reads with less than 60% of high quality bases (Phred score ≥ 20) were deleted; (3) 3′ end of reads were trimmed to the first high quality base. The subsequent high quality reads were further mapped to some cereal genomes (*Oryza sativa, Beijing Rice indica* and *Triticum aestivum*) by SOAPaligner (version 2.21), any hit associated with the reads and their mated reads were removed. Such filtered reads were admitted for the next steps of the analysis.

#### Species taxonomic assignment and abundance calculation

Clean paired-end reads were aligned to microbial genomes, available on NCBI and Genebank. A valid hit took place only if a read and its mated read mapped the same contig of a bacterial genome. The abundance was calculated with a previous study^38^, and each genome length was adjusted by subtracting the product of gap number and average insert length from the sum of contig lengths of the same genome.

#### De novo assembly of Illumina high quality reads

*De bruijn*-graph-based assembler SOAPdenovo (version 2.04) were employed to assemble short reads with parameters ‘-M 3 -u -L 100 -d 1 -F’. *k*-mers, varying from 39 to 59 by 4, were tested for each sample. The resulting scaffolds were cut into contigs at ambiguous Ns and only contigs longer than 500 bp were saved. N50 were calculated for contigs of different k-mers and only the contigs of largest N50 assembly were attributed to a sample. In order to utilize as many reads as possible, the unassembled reads from the same batch of rice wine fermentation were merged for a second assembly using only one *k-*mer 59^38^. All these contigs were applied for gene prediction.

#### Removing cereal contigs

Newly assembled contigs were compared with cereal genomes by blat (v. 35 × 1). The contigs, having ≥90% coverage and ≥95% identity, were considered to belong to cereal genomes and removed.

#### Gene prediction and determining contig origin

Since a contig might result from either eukaryotes or prokaryotes, which involved different software for gene prediction, it was necessary to classify contigs into either of the two domains. August (version 2.5.5) and MetaGeneMark (version 3.25) were employed for gene prediction of fungi and bacteria, respectively. Because the prediction of August needs a species genome as model, several species were tested, the predicted results with the best probability were chosen. Three steps were performed to separate fungal contigs from bacterial ones, as detailed in the follows: (1) Contigs that were gene-predictable by only one software were classified into the software associated domain. (2) Contigs, predicted by both the software, were compared with bacterial and fungal genomes using blat, contigs were assigned to a tax if (a) the coverage ≥90% and identity ≥95% or (b) the overlap between assembled contigs and genome contigs was larger than 100 bp with the identity ≥95%. If there were several assignments, the best one was preferred. (3) As for the rest predictable contigs, their correspondent protein sequences were annotated according to the NR database, a protein was assigned if E value was smaller than 1e-10. As long as at least 40% of genes on an assembled contig could be assigned to the same domain, that contig was thought to belong to that domain. In case of a contig still having ambiguous origin, it was distributed into a domain that generates a better average score in the output format of blast m8.

#### Gene catalogue

Removing redundancy was performed respectively on both bacterial and fungal redundant CDS catalogues. Sequences with ≥95% identity and 90% coverage were thought to be redundant and the longer one was left. Therefore, two CDS catalogues were obtained respectively for bacteria and fungi. These two CDS catalogue were merged into a catalogue note C{cds}. Furthermore, We got two others catalogues: (1) catalogue, consisting of non-redundant bacterial CDS catalogue and non-redundant fungal CDS-correspondent transcript catalogue, noted C{trans} and (2) catalogue, comprised of both non- redundant CDS-correspondent protein sequences, noted C{P}.

#### Gene profiling table

Clean paired reads were mapped to C{trans}, the formula of calculation of gene abundance was related to Qin *et al.*^38^.

#### QS (Quorum Sensing)

A local QS database was constructed by searching keywords about QS on UniProtKB^39^. The keywords were “accessory gene regulator”, “quorum sensing” or “autoinducer”. The resulting 44,778 protein sequences were dereplicated and the sequences were classified into 8 groups in the light of function of proteins. The groups includes “Autoinducer”, “Autoinducer_producer”, “Autoinducer_receptor”, “Decomposer”, “Effector”, “Regulator”, “Transporter” and “unclear”. C{P} was annotated to QS, An effective hit took place as long as the score ≥60 and E value < 1e-5^40^. As for the one with multiple hits, the best one was taken.

#### Antibiotic resistance gene analysis

C{P} was compared with ARDB (Antibiotic Resistant Database) using ARDB’s own pipeline and 80% identity threshold^41^ was selected for a valid hit.

#### KEGG and pathway annotation

C{P} was also annotated by KEGG (Kyoto Encyclopedia Genes and Genomes), using the same methods as QS. Gene abundances were compared between samples. A gene was thought significantly more abundant in one sample than in the other if the gene in one sample is tenfold as much as in the other. These markedly different genes were functionally marked on KEGG pathway.

## Additional Information

**How to cite this article**: Hong, X. *et al.* Metagenomic sequencing reveals the relationship between microbiota composition and quality of Chinese Rice Wine. *Sci. Rep.* **6**, 26621; doi: 10.1038/srep26621 (2016).

## Supplementary Material

Supplementary Information

Supplementary Table123
