# Supplementary material for: Metagenomic sequencing reveals the relationship between microbiota composition and quality of Chinese Rice Wine
Source: Sci Rep. 2016 May 31;6:26621. doi: 10.1038/srep26621 (PMC4886530; doi:10.1038/srep26621)
Supplement: Supplementary Information [file srep26621-s1.doc]

**Supplemental Information**

**Metagenomic sequencing reveals the relationship between microbiota composition and quality of Chinese Rice Wine**

Xutao Hong^1,2,*^, Jing Chen^3*^, Lin Liu^4,5*^, Huan Wu^3^, Haiqin Tan^1^, Guangfa Xie^6^, Qian Xu^3^, , Huijun Zou^6^, Wenjing Yu^1^, Lan Wang^4^&Nan Qin^4,5^

^1^ Zhejiang-California International Nanosystem Institute, Zhejiang University, 866 Yuhangtang Road, Hangzhou 310058, China

^2^ College of Life Sciences, Zhejiang University, Hangzhou 310058, People’s Republic of China

^3^ Realbio Genomics Institute, Shanghai, 200050, China

^4^ State Key Laboratory for Diagnosis and Treatment of Infectious Disease, The First Affiliated Hospital, College of Medicine, Zhejiang University, Hangzhou, 310003, China

^5^ Collaborative Innovation Center for Diagnosis and Treatment of Infectious Diseases, Zhejiang University, Hangzhou, 310003, China

^6^ National Engineering Research Center for Chinese Rice Wine, Zhejiang Guyuelongshan Shaoxing Rice Wine Co.Ltd., Shaoxing, Zhejiang 312000, China

*These authors contributed equally to this work.

Correspondence and requests for materials should be addressed to Nan Qin (email: nqin@zju.edu.cn)

**Supplemental Figure Legends**

**Supplementary Figure 1. PCoA on 16S of yeast starter on genus level.**

The panel is colored by sample qualities : qualities poor(green), intermediate (grey), good (red), perfect (blue).

**Supplementary Figure 2.** Bacterial composition of 16S of 110 samples on genus level.

The prefixes “k__”, “p__”, “c__”, “o__”, “f__” indicate OTUs only annotated to the level of kingdom, phylum, class, order or family.

The top of the panel is the label of sample qualities, The bottom is group information.

**Supplementary Figure 3. Top10 taxonomic composition calculated by different methods.**

(a) Bacterial composition of 16S. (b) Bacterial composition of metagenomics. (c) Fungal composition of ITS. (d) Fungal composition of metagenomics

**Supplementary Figure 4. Abundance of antibiotic resistant genes differs between batches.**

(a) Total abundance of AR genes varying with the fermentation time. Difference of significance between batches was test by Wilcoxon test (*≤ 0.05 ). (b) Heatmap diagram shows the abundance of the first 30 most abundant AR types. AR types are arranged in an ascending of average abundance.

**Supplemental Tables**

**Supplementary table: sample information and sequencing data summary.**

**
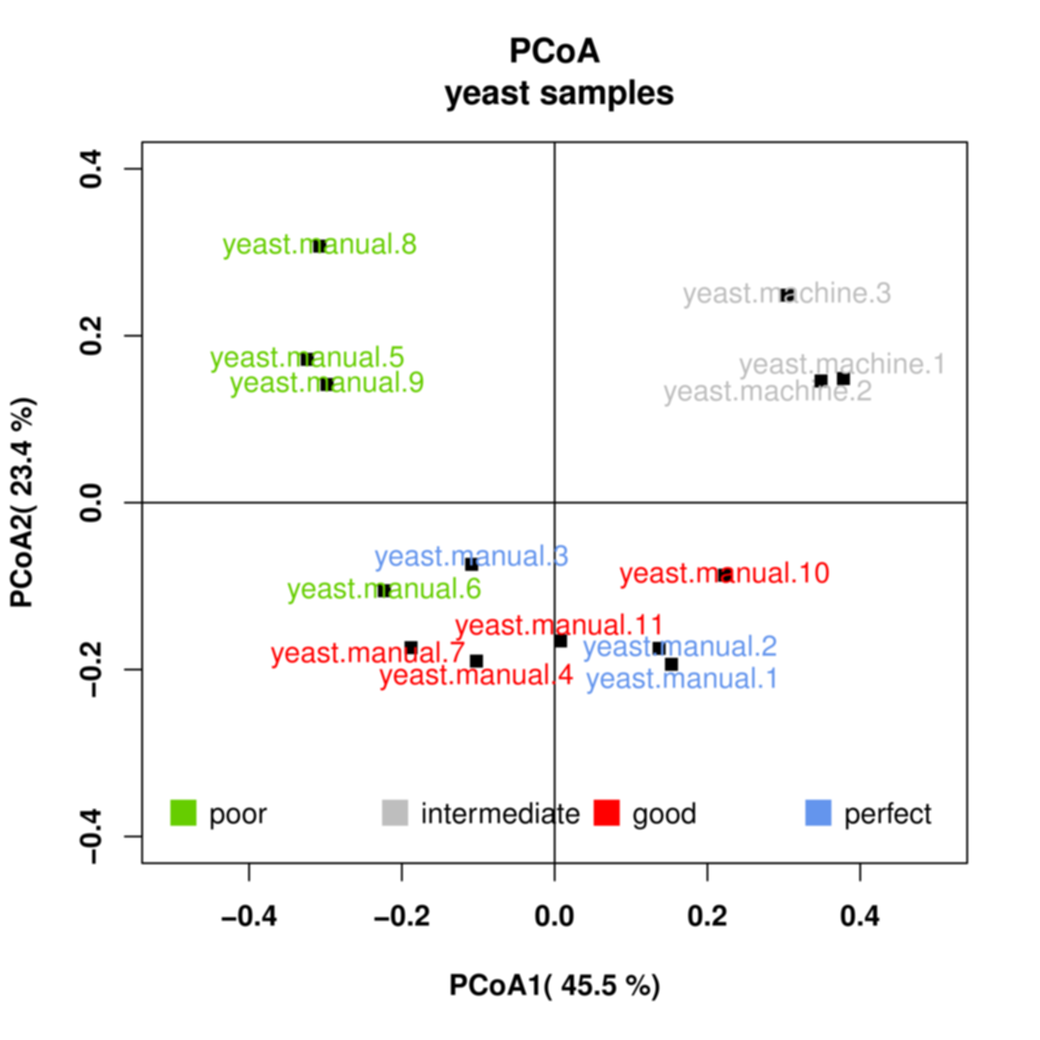
**

**
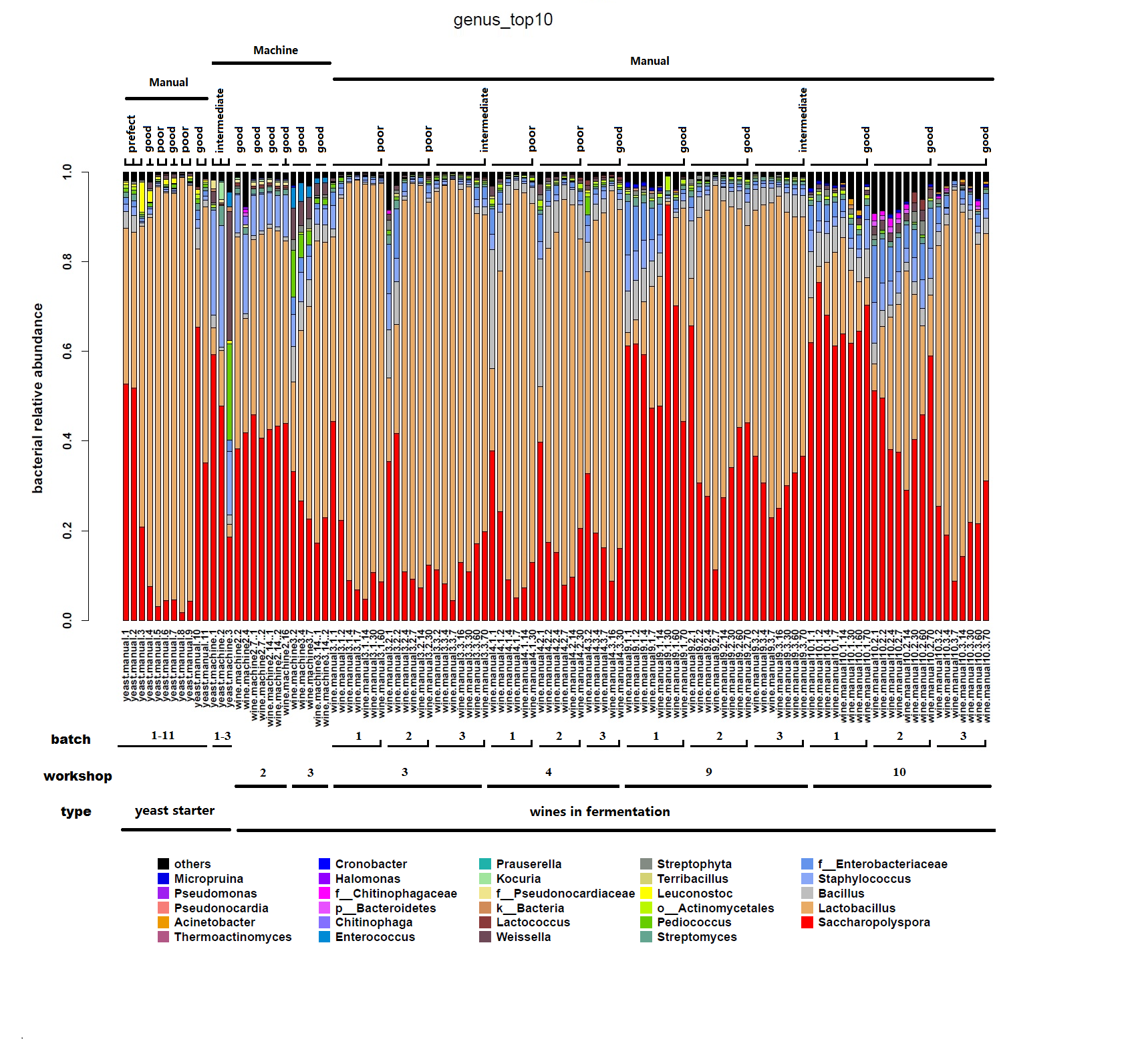
**

**
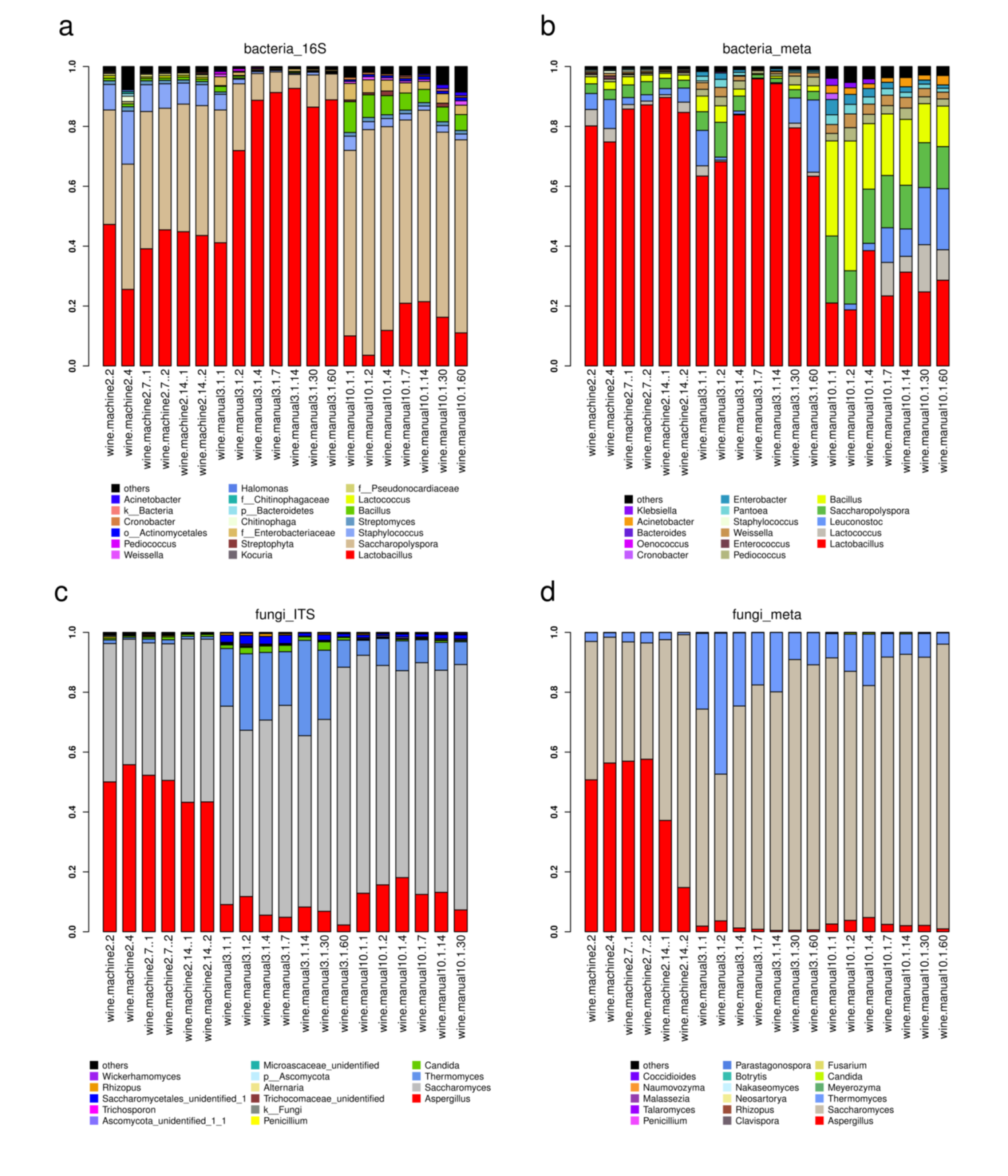
**

**
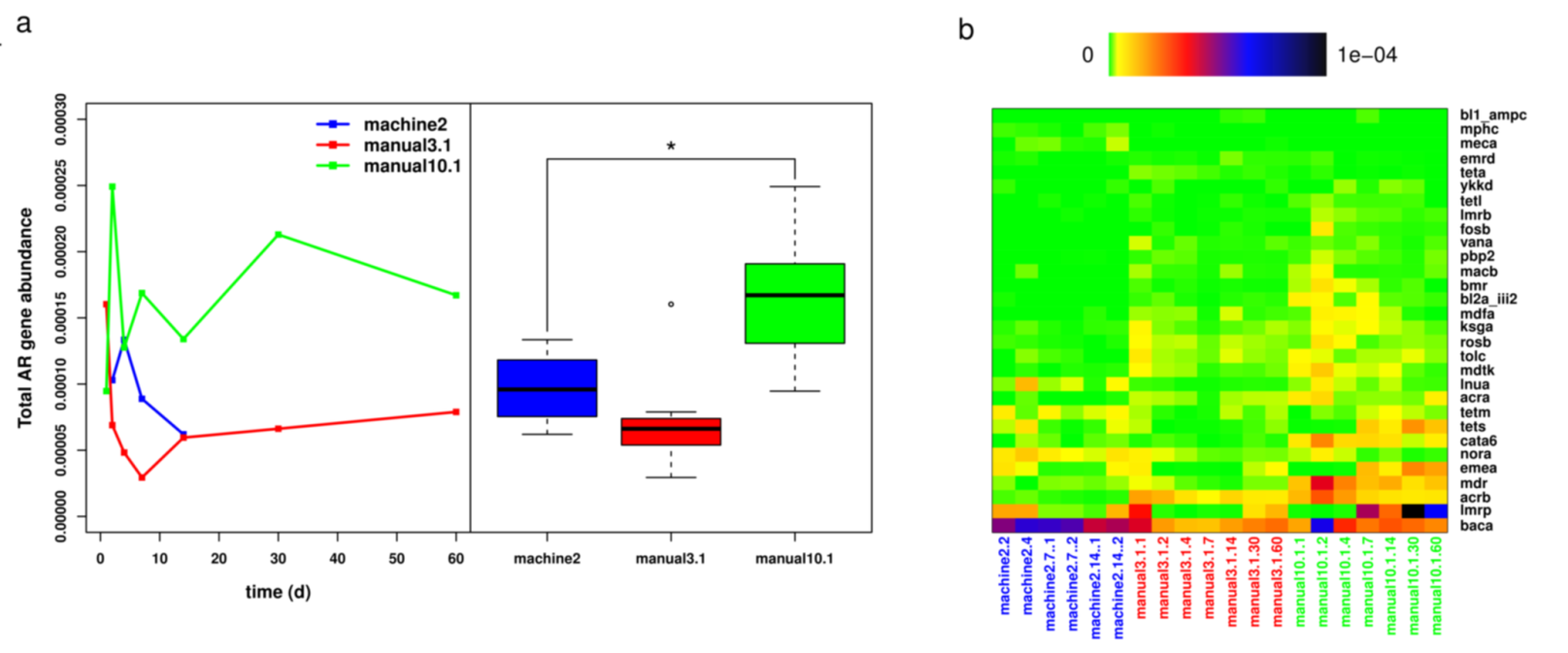
**
